# Are Long-Distance Walks Therapeutic? A Systematic Scoping Review of the Conceptualization of Long-Distance Walking and Its Relation to Mental Health

**DOI:** 10.3390/ijerph18157741

**Published:** 2021-07-21

**Authors:** Martin Mau, Anders Aaby, Søren Harnow Klausen, Kirsten Kaya Roessler

**Affiliations:** 1 Department of Psychology, University of Southern Denmark, 5230 Odense, Denmark; aaaby@health.sdu.dk (A.A.); kroessler@health.sdu.dk (K.K.R.); 2 Health, Social Work and Welfare Research, UCL University College, 5230 Odense, Denmark; 3 Health Sciences Research Centre, UCL University College, 5230 Odense, Denmark; 4 Specialized Hospital for Polio and Accident Victims, 2610 Rødovre, Denmark; 5 Department for the Study of Culture, University of Southern Denmark, 5230 Odense, Denmark; harnow@sdu.dk

**Keywords:** long-distance walking, hiking, pilgrimage, physical activity, walking, mental health, well-being, distress, nature

## Abstract

Long-distance walking is an ancient activity practiced across cultures for many reasons, including the improvement of one’s health. It has even been suggested that long-distance walking may be considered a form of psychotherapy. This scoping review examined the relationship between long-distance walking and mental health among adults. Publication trends and definitions were also examined, and the reason why long-distance walking may have therapeutic effects was discussed. Systematic searches in three online databases were performed using a selection of long-distance walking terms. Both quantitative and qualitative studies were included if they examined associations between long-distance walking and mental health in an adult population. Mental health was conceptualized in broad terms, including descriptions of mental states as well as more specific measurements or notions of mental health. A total of 8557 records were screened and 26 studies were included, out of which 15 were quantitative, 9 were qualitative, and 2 were mixed. The findings showed that long-distance walking was positively related to mental health. This was most consistent with regard to emotional distress compared to somewhat inconsistent findings regarding well-being. Therefore, long-distance walking may be more appropriately used to counter some personal or emotional struggle rather than to achieve hedonic pleasure.

## 1. Introduction

Long-distance walking has been practiced through the ages and across many cultures. Today, long-distance walking has become widely popular, and its popularity has been motivated by a host of different reasons related to notions of mental, physical, or spiritual health. This has led to long-established walking routes seeing an increasing number of visitors, such as the Camino de Santiago for instance, where walkers have tripled over a ten-year period, reaching 280,000 walkers in 2016 [1]. There are several purposes that motivate people to go on long-distance walks [2]. Physical health may be one motivation, as long-distance walking may lead to decreased blood pressure, an enhanced immune system, and more [3]. Another motivation is that long-distance walking may have a therapeutic effect.

One line of studies focusing on notions such as well-being and quality of life also suggests a therapeutic function of long-distance walking. A review focusing on outdoor recreation, including hiking, suggested that this may be viewed as a therapeutic medium by enhancing the quality of life for people with enduring mental health problems [4]. By combining the health benefits of physical activity and time spent in nature (i.e., “green exercise”), walking can be viewed as a readily available form of therapy [3,5]. A number of studies explain their findings through the synergistic effects of the benefits associated with walking and with exposure to nature (e.g., see [6]). Furthermore, neuroimaging studies show that walking through natural environments as compared to walking in urban environments may lead to reduced neural activity in an area of the brain associated with mental illness [7]. Similarly, there is a growing body of literature revealing the harmonizing effect of nature exposure on physiological stress reactions [8]. In parallel to the positive effects of nature exposure, the landscapes themselves may also influence the presumed therapeutic effect. Therapeutic landscapes are places where the visitors may experience “the promise and possibility of more restful psychosocial states” ([9] p. 35, in: [10]). The stillness associated with certain landscapes can enable the visitors to experience health-related effects [11] and may even stretch their perception of time [12].

In summary, long-distance walking might be a low-cost intervention to promote mental health [3]. It is highly accessible as the intensity of long-distance walking can be adjusted to the individual’s physical capacity [13]. There is no consensus as to what exactly constitutes a long-distance walk. Most commonly, it is defined as a walk of a certain distance (e.g., 30 km) or of a certain stretch of time (e.g., lasting several days) [14,15]. Tramping can be considered synonymous with long-distance walking, as this also implies walking for leisure for prolonged periods of time [16]. Although a pilgrimage has certain idiosyncrasies, it can be considered to fall under the generic term of long-distance walking.

A pilgrimage walk may provide “medicine for the soul”, relieving pain or filling a spiritual void that one might be experiencing [17]. Traditionally, the pilgrimage was based on religious affiliation, whereas today, secularized pilgrimages have emerged, and there seems to be no contrast between pilgrimage and modernity [18]. In fact, rather than displacing them, modernity has popularized pilgrimage walks and provided new interpretations with underlying meanings [19]. Indeed, because of modernity, people may now walk as a way of negating the impact of life in modern societies [20,21]. Considering these changes, defining pilgrimages is complex. From one perspective, a pilgrimage is viewed as a pursuit or quest associated with religious belief or a notion of what is sacred [22]. Such an understanding, however, emphasizes the oxymoronic character of the so-called secularized pilgrimages by necessitating some form of belief. In order to encompass the pilgrimages that are not based on belief, a pilgrimage may more simply be defined as a “journey redolent with meaning” [23] (p. 36). Such meaning *may* be based on belief but may also revolve around existential crises or notions of personal development. Egan [24] uses the term “the body as a memorial” to explain how the physical strain that the long-distance walkers expose themselves to may be chosen to express the personal struggles they are going through. The “wounded soul” of the walker may attain a more concrete form in the “wounded body” [24], and the pain associated with long-distance walking may become a vicarious expression of the pain one might be feeling psychologically. It has been suggested that the therapeutic pattern associated with long-distance walking is constituted by two elements: a realization of personal wounds or missing elements in one’s daily life, as explained by Egan [24], but also experiences of renewal or transformation [17]. Such transformation may be related to several different types of psychological change, including views of other people, feelings of competency, and empathy [25].

Although a number of explanations have been proposed and a wealth of empirical studies has been conducted, the effects of long-distance walking on mental health have not yet been systematically reviewed. A review of the research field is necessary to examine whether and why long-distance walking may be therapeutic, viewed through the lens of empirical research in the field. It will also provide a solid framework for discussing how long-distance walking should be defined as the current definitions are vague, unclear, and not universally adopted across studies. Due to the diversity of definitions and methodology, a scoping review with a systematic literature search is appropriate as its purpose is to map a research field that has not been reviewed comprehensively before [26].

The aim of this scoping review was to provide an answer to the question of how long-distance walking is related to mental health among adults. To answer this broad question, five specific research questions were developed to guide the review:

1. How has long-distance walking been defined?
2. How has long-distance walking been examined across time and place?
3. What effects does long-distance walking have on the mental health or states of adults?
4. Which aspects of the walk are associated with these effects?
5. Which theories are currently used to explain these effects?

## 2. Materials and Methods

This review is based on the scoping review process proposed by Arksey and O’Malley [26], updated by Levac et al. [27], and includes five steps: (1) identifying the research question, (2) identifying relevant studies, (3) selecting studies, (4) charting the data, and (5) collating, summarizing, and reporting the results. The review was reported in accordance with the PRISMA guideline extension for scoping reviews [28]. The protocol was registered in the Open Science Framework in February 2021 and is available online through https://osf.io/cnhys/ (accessed on 16 July 2021).

### 2.1. Search Strategy

Search terms were identified by the authors in cooperation with a research librarian. Pilot searches were conducted to ensure exhaustiveness. These pilot searches showed that a search algorithm focused on the term “long-distance walking” and synonyms thereof would retrieve a manageable number of records. The search algorithm thus comprised of a selection of long-distance walking terms used elsewhere in the literature with no other specifiers added [14,29].

A systematic search was conducted on 3 February 2021, in three online databases (Medline, PsycINFO, and Embase). Two limitations were added to exclude animal studies and conference abstracts. Supplementary searches were conducted in Scopus and Google Scholar. In Scopus, an additional search term was added to the search algorithm (“mental”), and in Google Scholar, the first 100 hits for each search term were screened. In addition to these database searches, one reviewer (MM) screened the reference lists of included studies and all studies citing the included studies. The latter was identified using Google Scholar. The complete list of search terms is presented in Table 1, and the search algorithm can be found in Appendix A and Appendix B.

Eligibility criteria were developed prior to screening. Peer-reviewed quantitative and qualitative studies with original, empirical data examining how long-distance walking was related to mental health or mental states were included. Only studies with a population of adults (18+ years of age) and published in English, Danish, Norwegian, or Swedish were included. Reviews, protocols, doctoral dissertations, masters or bachelors theses, editorials, and conference abstracts were excluded.

Mental health was conceptualized in broad terms, including both qualitative descriptions of mental states and more specific measurements or notions of mental health, either as questionnaires or biological markers. Studies on cognitive capabilities were excluded, as this was not considered to be the same as mental health. Long-distance walking was also broadly defined and no limitations were put on distance, duration, or setting (walks taking place in nature as well as urban settings were included). If the authors of a study characterized the walk as a long-distance walk, hike, trek, pilgrimage, or tramp (see Table 1), the study was considered relevant to this review. As a pilgrimage may include a range of activities other than walking (e.g., see [30]), only studies mentioning the specific relations between walking and mental health were included. Similarly, a study was excluded when results were not reported for the long-distance walk in isolation but in association with other outdoor activities, e.g., mountain climbing. Lastly, studies on exercise tests or those where long-distance walking was part of a job, e.g., as a porter, were excluded. See Appendix C for details on the inclusion and exclusion criteria.

### 2.2. Identifying Relevant Studies

First, all identified records were imported into Endnote wherein duplicates were removed. All unique records were then uploaded to the online screening tool Covidence [31] enabling the two raters (MM and AA) to independently assess the studies for relevance according to the eligibility criteria. After this, studies were screened based on titles and abstracts (step 1). Studies that could not be excluded based on their title and abstract alone were subsequently screened in their entirety (step 2). At this point, the reasons for exclusion were noted. At each step, the two raters discussed any disagreements, and a third author (KKR) was consulted in cases where disagreements could not be resolved.

### 2.3. Charting the Data

A data charting form was created based on the research question and specific objectives guiding this review. Descriptive information of the studies—including the year and place of publication, population examined, distance, duration, walk setting, study design, and notion of mental health—was extracted and summarized in Appendix D, Table A1. The key findings of the studies, including relationships between long-distance walking and mental health as well as potential explanations for these relationships put forth by the authors were extracted and summarized in Appendix D, Table A2.

### 2.4. Collating, Summarizing, and Reporting the Results

Following data extraction, the findings were analyzed manually through Thematic Analysis [32]. Thematic Analysis was chosen as it is considered a flexible approach, in which the researcher may attempt to inductively identify patterns in the data [33,34]. This analytic approach enabled the grouping of studies according to their design and findings, and to provide a summarized version of the published research. More specifically, the main author went through the six steps of the Thematic Analysis: (1) familiarization with data, (2) generating initial codes, (3) searching for themes, (4) reviewing of themes, (5) defining and naming themes, and finally, (6) producing the report, which, in this case, is the results section of this paper. The author did this for each of the questions presented in the results section, focusing on: notions of long-distance walking, notions of mental health, publication trends over time, and finally, the relationships between long-distance walking and mental health, and possible explanations underlying this relationship.

## 3. Results

**Figure 1:**
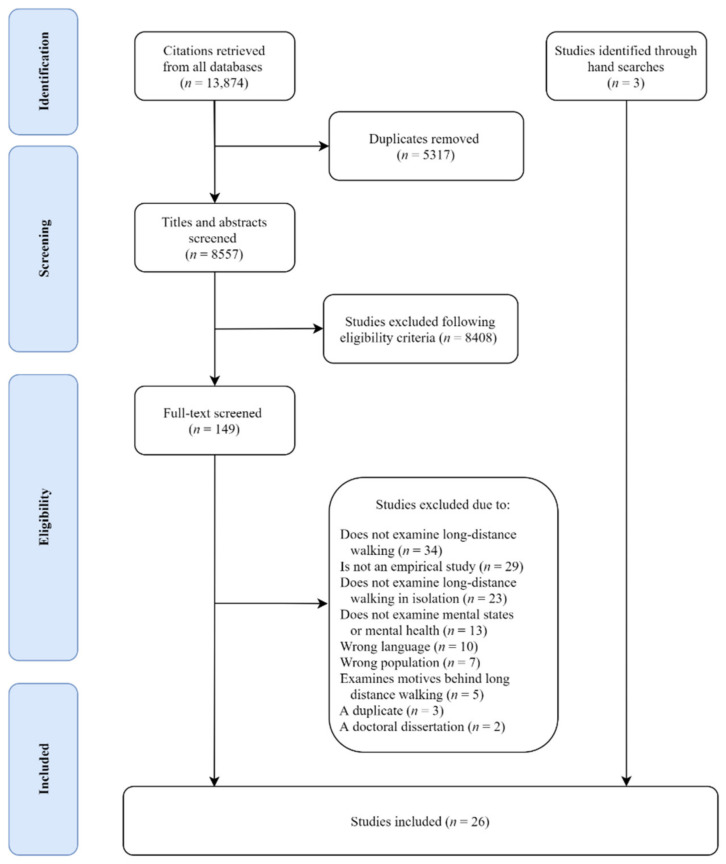
Flow chart illustrating the search process in a scoping review on long-distance walking and mental health.

The searches yielded 8557 references after duplicate removal (see Figure 1). A total of 26 studies were included (for an overview of included studies, see Appendix D, Table A1 and Table A2).

### 3.1. How Was Long-Distance Walking Defined?

Long-distance walking was often defined in terms of how much time it took, sometimes in terms of the distance walked, and sometimes as both (see Appendix D, Table A1). To describe the walk, 13 studies reported only time, whereas five reported distance, five reported both time and distance, and three reported neither time nor distance.

Duration and distance varied and could be divided into three groups. The first group, called the “short long-distance walk” (short LDW), included eight studies that examined walks that lasted for one day or were 12 km at most. The second group, called the “long long-distance walk” (long LDW), included 12 studies that examined walks that lasted multiple days or were 100 km or more. The last group included six studies that examined walks of unspecified duration; they either did not provide information or were unspecific, e.g., those that ranged from hours to days. The walks were usually completed either partly or entirely in groups, and they usually took place in nature, e.g., in forests or mountainous areas.

### 3.2. What Were the Publication Trends for Time and Place?

A large majority of the studies were published during the last 10 years (23 out of 26), and about half of these within the last five years (see Appendix D, Table A1 for a complete overview of study characteristics).

The country of study and study design varied, with 18 of the studies coming from a European country. Four were from North America and the remaining were from Turkey, Australia, China, and India. There were more quantitative studies (*n* = 15) than qualitative studies (*n* = 9), while two used both quantitative and qualitative methods. Of the quantitative studies, all used self-completion questionnaires, except for three studies that used a biological marker either exclusively or in conjunction with a self-completion questionnaire. Five of the quantitative studies were cross-sectional and the remaining were longitudinal. Quantitative methods were used to examine both short LDW and long LDW. Of the qualitative studies, seven collected data using interviews (focus group, individual, unstructured, semi-structured), one study used an ethnographic method, and one study used an open-ended questionnaire. Qualitative methods were mainly used to examine long LDW.

There were not any trends regarding population sex or age, with the studies including participants who were in their 30s up to those in their 80s. Most participants were already long-distance walkers as contact was achieved through approaching walkers on paths, chain-sampling, or walking clubs. A total of 7 out of the 26 studies focused on a specific group, e.g., psychiatric patients or cancer survivors. These specific groups were examined in both qualitative and quantitative studies.

### 3.3. Which Indicators of Mental Health Were Examined?

The notions of mental health or mental states varied and depended on the study design. The studies that used a quantitative method could be divided into two groups. The first group comprised ten studies that used measurements related to some aspect of well-being, including life satisfaction, quality of life, subjective well-being, flow, enjoyment, or mindfulness. This group consisted of studies examining both short LDW, long LDW, and walks of unspecified duration. The second group comprised seven studies that measured aspects of emotional distress, including stress, depression or suicidality, anxiety, and overall distress. This group consisted of studies mainly examining short LDW. The one remaining quantitative study focused on affective responses, both positive and negative, thus not fitting into either of these two groups [35].

Regarding studies that used a qualitative method (*n* = 11), the majority focused on how the walk influenced the participants’ general approach to life, without applying a more specific focus in the study. In this group of studies, participants usually went on long LDW (see Table 2). The remaining qualitative studies consisted of a mixed group of studies examining body awareness and identity, fear or anxiety, and mood. In summary, the included studies reported on a variety of indicators of mental health. Quantitative studies examined well-being and emotional distress, whereas qualitative studies focused on how the walk influenced the participants’ general approach to life along with other foci that were more specific.

### 3.4. Relationship between Long-Distance Walking and Mental Health

The included studies generally found that long-distance walking was related to the state or health indicator they were examining, with only a few exceptions (see Appendix D, Table A2). The studies that focused on well-being generally found that long-distance walking was related to higher well-being. Five of these studies were cross-sectional and five were longitudinal. Among the cross-sectional studies, all found a positive association. Among the longitudinal, the association was more inconsistent; three found an association while two did not. The two studies that did not find an association focused on notions of well-being [39,45], whereas the three that did, focused on enjoyment, quality of life, and mindfulness [38,43,56].

Seven of the studies provided explanations as to why long-distance walking might have affected well-being. These varied from focusing on notions of gaining a positive outlook, how participants might feel younger as a result of the walk, how walks might lead to a feeling of freedom, and how there might be overlaps between long-distance walking and meditation practices. The most common explanation focused on a synergistic effect of positive outcomes from walking and from exposure to nature. This is also known as “green exercise” and builds on the notion that the gains from physical exercise are amplified when performed in nature, which may have restorative effects in the context of mental well-being.

Studies that focused on emotional distress generally found a positive effect of long-distance walking. No effect of study design was identified as they were all longitudinal. The only study that did not find a positive effect found that long-distance walking did *not* have a negative effect [40]. One study [39] examined both measures of emotional distress and well-being and found that long-distance walking was not related to well-being but was related to a significant reduction in some measures of emotional distress. This, however, depended on the measure of emotional distress, finding non-significant reductions in depression and generalized anxiety, but significant reductions with regards to stress and state anxiety. However, one other study, which also examined depression, found a significant reduction after long-distance walking. Stress was examined by four of the studies, either through questionnaires or biological markers, all of which found significant reductions after long-distance walking.

Four of these studies provided explanations on why the walk might have had this effect. The common explanation built on the notion of “green exercise”, combining the positive effects of the walk and nature exposure. However, one study questioned the effect of nature as they found no difference between mountain hiking and treadmill walking in their measure of emotional distress [41].

The majority of qualitative studies focused on the participants’ general approach to life and found that long-distance walking enabled a focus on self, thus giving time to, e.g., discovering oneself or managing emotionally difficult experiences. These studies also focused on how the walks provided a sense of strength in the form of self-efficacy, attaining a sense of capability, or independence. Disengagement was also a common theme across studies in this group. Within these studies, the effect of the walk was mainly explained through different theories within a Positive Psychology framework such as the broaden-and-build theory [59] and the flow theory [60]. Five studies focused on specific foci and found that long-distance walking had a favorable effect on body awareness, fear, anxiety, and mood. To understand this influence, these studies applied different theoretical frameworks such as body awareness theories [61] and the flow theory [60].

## 4. Discussion

The aim of this scoping review was to examine how long-distance walking was related to mental health. To answer this broad question, five specific research questions were developed, which will each be addressed below. First, regarding definitions, long-distance walking appeared to be defined more often in terms of time rather than distance. How much time the participants spent varied greatly; from walks lasting one day or less to walks lasting several weeks or months. Secondly, regarding publication trends and designs, a broad range of methods was used, including quantitative and qualitative, intervention and observational, and cross-sectional and longitudinal studies. Furthermore, different samples were included, with both young and old as well as ill and non-ill populations being explored; the participants were recruited from psychiatric treatment facilities or simply from hiking paths. Recruiting through snowball sampling was the most typical method of inclusion. The heterogeneity in participants across studies suggests that long-distance walking is not only relevant to practiced walkers but may be beneficial for both ill and non-ill, young and old, men and women, as well as in a group and individually.

Conversely, the research field was homogenous in other respects. Almost all studies were published within the last 20 years, and the large majority were European. Although long-distance walking is an ancient activity practiced for centuries across cultures (e.g., see [24]), the scientific research is recent and originates mainly in Europe. This might have created bias in the findings of this review and suggests that there may be different and more culture-dependent forms of long-distance walking that are not reviewed here. This bias might be a consequence of the eligibility criteria where studies were excluded if the effect of long-distance walking could not be isolated from other activities such as religious rituals. This exclusion was necessary in order to investigate the research question of this review, consequently selecting studies on long-distance walking without a religious component, which might be more related to modern western culture. Future reviews could focus specifically on examining differences between long-distance walks with and without religious activities.

Thirdly, the findings collectively suggested that long-distance walking may be a remedy against mental health issues. In fact, even short LDW showed beneficial effects on various aspects of mental distress such as stress, depression, and anxiety. Whether longer walks yield greater effects on mental health was not possible to determine decisively. However, the qualitative studies tentatively suggested that long LDW may provide more significant effects than short LDW on mental distress. These studies emphasized aspects such as time and disengagement, which, although subjectively perceived, may be related to the duration of the walk. In contrast to the studies on mental distress, the same beneficial effects of long-distance walking were not as consistently found when considering aspects of well-being. Though most cross-sectional studies found a positive association, the longitudinal studies were conflicting.

Lastly, the studies generally leaned on the same two branches of theories to explain the beneficial effects of long-distance walking: different sub-theories related to Positive Psychology [62] (e.g., flow theory, broaden and build theory [63]), or the notion of “green exercise”. The theory of “green exercise” builds on a coupling of research findings and theories related to the beneficial effects of exercise and those of exposure to nature. These were used to explain the effects of long-distance walking on mental distress and well-being, although the effect on well-being was, as previously described, inconsistent. Further in-depth theoretical accounts linked closely to empirical findings are warranted.

### 4.1. The Role of Long-Distance Walking: Well-Being or Meaning?

There are several suggestions as to why long-distance walking may be therapeutic. These theories were used in a rather generic way in the included studies and were mostly not related to a specific aspect of the walk. There were, however, two exceptions to this. One study examined the effects of anthropogenic elements (e.g., buildings) and did not find any additional benefit of having fewer anthropogenic elements in the walk [42]. Another study examined the effect of nature by having a control group use a treadmill. Similarly, this study did not find any additional benefit of walking in nature as opposed to using a treadmill [41]. Both studies noted that they could have missed some of the benefits of exposure to nature and are therefore not able to rule out an effect of nature exposure.

One of the key findings of this review was that long-distance walking seemed to be consistently related to lower mental distress in longitudinal studies. In contrast, the evidence for an association between long-distance walking and well-being was conflicting in the longitudinal studies. This makes it uncertain whether long-distance walking may facilitate a change in well-being rather than simply be associated with a relatively high baseline level. This, we believe, supports the assertion that long-distance walking may be a “journey redolent with meaning” [23] (p. 36), emphasizing the deeper and perhaps more profound mental benefits provided by the walks, rather than hedonistic well-being. It may thus be hypothesized that, just as therapy is not necessarily pleasurable, the same can be said of long-distance walking. The time available as well as the opportunity to focus on and contemplate difficult experiences without distractions (e.g., from social obligations) may provide a space that is at times difficult or tiring, but nevertheless helpful.

How far the studies can be taken to show that long-distance walking does not increase well-being, is, however, debatable. It depends on how broadly or narrowly one understands well-being. One view is this ‘hedonic’ notion of well-being, consisting of life satisfaction or simply pleasurable experiences. In a broader but still subjectivist view, a reduction of emotional distress would itself count as an instance of increased well-being because it is seen as consisting in a predominance of positive over negative experiences and emotions. This is indeed a standard view in more theoretically informed studies of well-being (e.g., [63,64,65]). Eliminating or controlling negative experiences arguably makes one better off, even if it is not perceived as positively pleasurable.

Moreover, there are other understandings of this state that do not juxtapose well-being with meaning and personal development. Conceptions of well-being as human flourishing take meaning and self-realization to be its most central components [66]. Hence, while the studies may be taken to indicate that long-distance walking is not particularly conducive to immediate or vivid pleasure (which is perhaps not that surprising, given that it is a strenuous and physically demanding activity, or that walkers attend to difficult emotions), this does not rule out the notion that long-distance walking could still be conducive to well-being in a more comprehensive or profound sense, and the studies may even be said to provide indirect evidence of this.

A similar review to this one was recently conducted on the psychology of long-distance running [67]. In this review, it was concluded that the effects of long-distance running included an increase in fatigue and a decrease in vigor and tension. In parallel to the discussion above on the somewhat inconsistent findings on long-distance walking and well-being, the authors suggest that this may stem from the prolonged endurance efforts, which is part of long-distance running. However, whether there is a similar therapeutic potential associated with long-distance running to that associated with long-distance walking still needs to be addressed.

### 4.2. Strengths and Limitations

This scoping review has several strengths and limitations. The major strength was the literature search, which was thorough and comprehensive with a broad conceptualization of mental health that included all studies examining mental states. Furthermore, it provided a broad understanding of long-distance walking, which was appropriate in a scoping review in order to map the research field.

Conversely, one of the major limitations was that grey literature was not considered. To provide a more scientifically sound discussion on our research question, only peer-reviewed studies were included. However, grey literature could have been relevant when examining publication trends over time, including which definitions of long-distance walking are being applied in the context of mental health. Another limitation pertains to the lack of quality assessment [27]. While this is generally not conducted in scoping reviews [27], it still adds potential bias to the review as studies with poor methodological quality are given the same weight as studies with good methodological quality. Lastly, studies on pilgrimages were largely excluded as the walk could not be isolated from other rituals. While this was necessary to answer the research question, it did limit the review to inadvertently focus on studies from western cultures.

## 5. Conclusions

This scoping review identified all empirical research examining the relations between long-distance walking and mental health. The main finding was a consistent positive relationship between long-distance walking and mental health. This was especially salient in terms of mental distress. Therefore, long-distance walking may be promising in the more general treatment of mental illness or distress. The qualitative studies suggested that the active components of the walk may include aspects such as time, a possibility to focus on oneself, and the possibility to find or show personal strength. In contrast to the very consistent findings on mental distress, the findings related to notions of well-being were somewhat more inconsistent. For this reason, long-distance walking may be defined as an activity “redolent with meaning”, used to counter some personal or emotional struggle rather than hedonic pleasure alone. However, as we did not assess the methodological quality of the included studies, it can be assumed that bias might have influenced the findings, which should therefore be interpreted accordingly.

The review also highlighted several important conceptual and methodological points: i) Long-distance walking was generally defined in terms of the time it took, ii) in the context of mental health, it is a relatively new research field consisting mainly of European studies, and iii) that it is a rather fragmented research field that uses a broad range of methods and includes a wide variety of populations.

To reach a stage where we can more firmly conclude whether or not long-distance walking may function as a form of psychotherapy, we suggest that future studies be conducted. This may be done through RCTs or *n* = 1 studies, with multiple measurements and preferably during a longer long-distance walk. We found that there was a great discrepancy between how long a long-distance walk was perceived to be. To identify what may make the long-distance walk unique in comparison to shorter walks or other forms of physical activity, we suggest an examination of walks of at least one full day.

## Figures and Tables

**Table 1:** The search strategy used in Medline, PsycINFO, and Embase for a scoping review on long-distance walking and mental health.

Main Term	Expanded Term
Long-distance walking	Long * adj3 walk * OR Far adj3 walk *
Trekking	Trek OR Treks OR Trekked OR Trekking OR Trekker
Hiking	Hike * OR Hiking
Pilgrimage	Pilgrim *
Tramping	Tramping OR Tramped OR Tramp

* = including all alternate endings of the term.

**Table 2:** Overview of included studies based on notions of mental health and definition of long-distance walk.

	Quantitative, Well-Being	Quantitative, Emotional Distress	Qualitative, General Approach to Life	Qualitative, Specific Foci
Short long-distance walk	[36,37,38,39]	[39,40,41,42]		[39]
Long long-distance walk	[43,44,45]	[46,47]	[15,48,49,50]	[51,52,53]
Unspecified duration	[54,55,56]		[21,57]	[58]

## Appendix A. Search Word Syntax for Main Search

### Appendix A.1. Ovid Medline(R) All <1946 to 01 February 2021>

**Table:**

1	(long * adj3 walk *).ti,ab.	1317
2	(far adj3 walk *).ti,ab.	80
3	(hike * or hiking).ti,ab.	1567
4	(trek or treks or trekked or trekking or trekker).ti,ab.	1271
5	Pilgrim *.ti,ab.	1308
6	(tramping or tramped or tramp).ti,ab.	1077
7	1 or 2 or 3 or 4 or 5 or 6	6576
8	exp animals/not humans.sh.	4,782,806
9	7 not 8	5710

* = including all alternate endings of the term.

### Appendix A.2. Embase Classic + Embase <1947 to 1 February 2021>

**Table:**

1	(long * adj3 walk *).ti,ab.	2007
2	(far adj3 walk *).ti,ab.	130
3	(hike * or hiking).ti,ab.	2090
4	(trek or treks or trekked or trekking or trekker).ti,ab.	1829
5	Pilgrim *.ti,ab.	1471
6	(tramping or tramped or tramp).ti,ab.	1628
7	1 or 2 or 3 or 4 or 5 or 6	9094
8	(exp animal/or animal experiment/ or nonhuman/) not (exp human/or human experiment/)	7,434,374
9	7 not 8	7375
10	limit 9 to conference abstract	1750
11	9 not 10	5625

* = including all alternate endings of the term.

### Appendix A.3. APA PsycInfo <1806 to Week 4 of January 2021>

**Table:**

1	(long * adj3 walk *).ti,ab.	281
2	(far adj3 walk *).ti,ab.	25
3	(hike * or hiking).ti,ab.	358
4	(trek or treks or trekked or trekking or trekker).ti,ab.	283
5	Pilgrim *.ti,ab.	684
6	(tramping or tramped or tramp).ti,ab.	54
7	1 or 2 or 3 or 4 or 5 or 6	1670
8	exp animals/not humans.sh.	355,003
9	7 not 8	1561

* = including all alternate endings of the term.

## Appendix B. Search Word Syntax for Supplementary Searches

### Appendix B.1. Scopus Search Word Syntax

**Table:**

1	TITLE-ABS-KEY (long * W/2 walk *)	2557
2	TITLE-ABS-KEY (far W/2 walk *)	1044
3	TITLE-ABS-KEY (hike * OR hiking)	7951
4	TITLE-ABS-KEY (trek OR treks OR trekked OR trekking OR trekker)	3381
5	TITLE-ABS-KEY (pilgrim *)	9241
6	TITLE-ABS-KEY (tramping OR tramped OR tramp)	2507
7	(TITLE-ABS-KEY (long * W/2 walk *)) OR (TITLE-ABS-KEY (far W/2 walk *)) OR (TITLE-ABS-KEY (hike* OR hiking)) OR (TITLE-ABS-KEY (trek OR treks OR trekked OR trekking OR trekker)) OR (TITLE-ABS-KEY (pilgrim *)) OR (TITLE-ABS-KEY (tramping OR tramped OR tramp))	26,455
8	TITLE-ABS-KEY (mental)	1,062,428
9	((TITLE-ABS-KEY (long * W/2 walk *)) OR (TITLE-ABS-KEY (far W/2 walk *)) OR (TITLE-ABS-KEY (hike * OR hiking)) OR (TITLE-ABS-KEY (trek OR treks OR trekked OR trekking OR trekker)) OR (TITLE-ABS-KEY (pilgrim *)) OR (TITLE-ABS-KEY (tramping OR tramped OR tramp))) AND (TITLE-ABS-KEY (mental))	371

* = including all alternate endings of the term.

### Appendix B.2. Google Scholar Search Word Syntax

The first 100 hits were screened for each of the main search terms:

(1) “long-distance walk”;
(2) “far walk”;
(3) Hike OR Hiker OR Hikes OR Hiking;
(4) Trek OR Treks OR Trekked OR Trekking OR Trekker;
(5) Pilgrim OR Pilgrimage OR Pilgrims;
(6) Tramping OR Tramped OR Tramp.

## Appendix C. Inclusion and Exclusion Criteria

Inclusion and exclusion criteria used to identify studies examining how long-distance walking is related to mental states among adults.

**Inclusion Criteria**	**Exclusion Criteria**
Original empirical data	Protocols, commentaries, editorials, conference abstracts, calls for research, letters to the editor, proceedings, book reviews, textbooks, replies from author, erratum, doctoral dissertations, master’s or bachelor’s theses, and opinion. Studies based on author’s reflections on own experiences.
Examines long-distance walking	Studies where the notion of long-distance walking is used in the context of exercise tests examining walking capabilities (e.g., 6 min walk test, 12 min walk test, long-distance corridor walk, shuttle-walk test), or studies where the notion of long-distance walking is used in the context of walks to school or as porters (i.e., where the walk is not viewed as a recreational activity). Studies on pilgrimage that do not include walking (only other modes of traveling or where walking is performed in combination with other forms of travel, and results are not presented for the walk specifically) or where the mode of travel is not specified. Studies on “walkers” or where data on “hikers” and “walkers” are mixed, but *include* studies where hikers/long-distance walkers, etc. are undertaking, e.g., “day-walks”.
Examines how long-distance walking was related to mental health	Studies on mountain/altitude/high-altitude sickness/illness, headache, etc., where the health aspect of the walk was described only in terms of somatic symptoms. Studies on long-distance walkers, where their mental health or mental states are not examined in association with the long-distance walk.
Adult population (18+ years)	
English, Danish, Swedish, or Norwegian language	

## Appendix D. Overview of 26 Studies on Long-Distance Walking and Mental Health

**Table A1:** Overview of study characteristics.

- Reference - 1st Author - Year of Publication - Country of Publication	Study Design (Quantitative: Cross-Sectional/Longitudinal, Qualitative: Retrospective/Prospective) [Control Groups?]	Study Population Characteristics: - Recruitment - N - Age - Sex Distribution	Past Experience with Long-Distance Walking	Setting of the Walk	Duration of Walk	Individual or Group Walk	Mental State Outcomes
- [36] - Clark - 1999 - USA	- Quantitative (self-completion questionnaires), cross-sectional	- Through senior citizen centers - 376 (33% were long walkers) - 65 and older - All female	- NA	- NA	- About 1 mile, at least 3 times/week	- NA	- Life satisfaction - Cognitive age
- [58] - Coble - 2003 - USA	- Qualitative (semi-structured interviews), retrospective	- Through chain sampling - 20 - Mean 34 years - 10 males	- Male participants had been hiking for 17 years on average, females for about 10 years	- Natural environments, especially the woods	- From 2 h to several days	- Individually	- Fears
- [43] - Den Breejen - 2007 - UK	- Quantitative (self-completion questionnaires), longitudinal	- By approaching walkers on the path - 15 - NA - 6 males	- Beginner–experienced	- The West Highland Way in Scotland	- 5–8 days	- Individually and in groups	- Enjoyment levels
- [15] - Crust - 2011 - UK	- Qualitative (unstructured interviews), retrospective	- Through purposive sampling - 6 - Mean 39.6 years - 4 males	- At least 3 years experience	- In nature, in the North of England	- 84–192 miles - 6–11 days	- In groups, unclear whether individually	- The psychological journey
- [54] - Ardahan - 2012 - Turkey	- Quantitative (self-completion questionnaires), cross-sectional	- Through clubs - 382 - Mean 39.92 years - NA	- NA	- NA	- NA	- NA	- Life satisfaction
- [46] - Sturm - 2012 - Austria	- Quantitative (self-completion questionnaires), longitudinal	- Through e-mail to former patients at a psychiatric clinic - 17 - NA - NA	- NA	- A mountainous area	- 12–26 days	- In groups	- Hopelessness - Depression - Suicide ideation
- [55] - Wöran - 2012 - Austria	- Quantitative (self-completion questionnaires), cross-sectional	- By contacting hikers in mountain huts on the trail - 369 - Mean 41.4 years - 47% female	- NA	- Alpine meadows, forests and rocks, the Austrian Salzkammergut	- A day trip, a half-day trip, or a trip lasting several days	- NA	- The experience of flow
- [48] - Saunders - 2013 - UK	- Qualitative (semi-structured interviews), retrospective	- Through clubs, websites, and chain-referral - 25 - 30+ - NA	- Novice–very experienced	- Most walks were nature-based treks	- 3 days–3 months	- In groups, unclear whether individually	- The transformative potential
- [49] - Olafsdottir - 2013 - Luxembourg	- Qualitative (ethnography), prospective	- Through participation in a guided trek - 9 - 31–58 years - 6 males	- Most were experienced	- A wilderness area	- 14 days	- In groups	- The relationships between humans and nature
- [40] - Neunhäuserer - 2013 - Austria	- Quantitative (blood markers), longitudinal	- Through an outpatient clinic with patients with high-risk suicidality - 17 - Mean 43.9 years - 4 males	- NA	- Alpine terrain	- 2–3 hikes per week for 9 weeks, 6–8 km per hike	- In groups	- Cytokines, associated with suicidality
- [37] - Wolf - 2014 - Australia	- Quantitative (questionnaire), cross-sectional	- By randomly intercepting visitors at track heads - 69 (371 in total) - NA - 66.2% male (total pop 56.6)	- The majority of participants hiked several times per month	- National parks, Australia	- 2.1 h/6.6 km on average	- Individually and in groups	- Well-being
- [53] - Mazzeschi - 2014 - Italy	- Quantitative (questionnaire), longitudinal, and - Qualitative (writings), prospective	- NA - 76 (36 normal weight, 40 overweight/obese) - Normal weight: Mean 54.2 years; Overweight/obese: Mean 58 years - NA	- Normal weight participants were habitual long-distance trekkers; Overweight/obese were trained before the trek	- Scenic footpaths between the cities of Ancona and Talamone	- Normal weight group: mean 9.1 days, 274 km; Overweight/obese group: mean 10.3 days, 308 km	- In groups	Quantitative: - Overall distress/Mood Qualitative: - The qualitative effect of the experience and the possible mechanisms through which physical activity influences mood
- [51] - Calsius - 2015 - Belgium	- Qualitative (focus-group interview), retrospective	- Through neurologists and physiotherapists, including participants with mild/moderate degree of neurological disability due to multiple sclerosis and high motivation - 9 - Median age 42 years - 3 males	- NA	- The Andes	- 5 days	- In groups	- Body awareness - Identity
- [41] - Niedermeier #1 - 2017 - Austria	- Quantitative (salivary cortisol), longitudinal	- Through website and e-mail announcements - 42 - Mean 32 years - 52% female	- NA	- A mountain hiking area, Innsbruck	- 12 km	- In groups	- Stress-related physiological responses
- [35] - Niedermeier #2 - 2017 - Austria	- Quantitative (self-completion questionnaires), longitudinal	- Through website and e-mail announcements - 42 - Mean 32 years - 52% female	- NA	- A mountain hiking area, Innsbruck	- 6 km	- In groups	- Affective responses
- [38] - Hepperger - 2017 - Austria	- Quantitative (self-completion questionnaires), longitudinal	- By contacting patients with knee osteoarthritis who had undergone total knee arthroplasty - 46 (25 in intervention group) - Mean 67 years - 60% female	- NA	- In nature in Innsbruckand surroundings.	- 3 month hiking programme, average 3.5 h, 2–3 times/week	- In groups	- Quality of life
- [57] - Eichberg - 2017 - Italy	- Qualitative (interviews)	- NA - 4 - Mean 80 years - 2 females	- Some were experienced, some inexperienced	- El Camino, a pilgrimage in Medjugorie, and a pilgrimage in Lourdes	- NA	- Individually and in groups	- Psychological wellness
- [44] - Yang - 2018 - China	- Quantitative (questionnaires distributed face-to-face), cross-sectional	- By approaching walkers on the path - 926 - 76% were under 35 years - 72.3% male	- NA	- Multiple environments, Shenzhen city	- About 100 km	- In groups	- Well-being
- [42] - Niedermeier - 2019 - Austria	- Quantitative (self-completion questionnaires, salivary cortisol), longitudinal	- Through public announcements on the web page of the Austrian Alpine Association - 52 measuring affective states, 44 measuring stress-related physiological responses - Mean 47 years - 57.7% female	- NA	- One mountain tour with anthropogenic elements and one without	- Both tours were approximately 7 km/3 h	- In groups	- Affective states- Stress-related physiological responses
- [45] - Sheldon - 2019 - USA	- Quantitative (self-completion questionnaires), longitudinal	- Through social media - 93 - Mean 37 years - 50 females, 1 transgender/other	- The majority had backpacking experience	- The pacific crest trail running along the mountainous spine of the U.S. West Coast	- Mean total miles walked: 1834	- NA	- Well-being
- [52] - Calsius - 2019 - Belgium	- Qualitative (focus-group interview), retrospective	- Through centres working with people with multiple sclerosis - 9 - 27–59 years - 6 females	- NA	- The Jordan Desert	- 10 days	- In groups	- Body awareness - Identity
- [50] - Jørgensen - 2020 - Norway	- Qualitative (open-ended questionnaires), prospective	- Through accommodations along the path - 53 - Mean 52 years - 30 females	- NA	- St. Olavs Route in Norway and Sweden	- The majority walked for more than two weeks, around 20 km per day; Max 42 days	- Individually and in groups	- Health-related processes and changes/effects
- [56] - Feliu-Soler - 2020 - Spain	- Quantitative (self-completion questionnaires), longitudinal	- Through associations, hostels, websites, and social media - 314 - NA - NA	- NA	- The Way of Saint James	- NA	- NA	- Mindfulness
- [39] - Lesser - 2020 - Canada	- Quantitative (self-completion questionnaires), longitudinal, and - Qualitative (semi-structured interviews), retrospective	- By contacting cancer survivors on social media, newspapers, poster advertisements, and the local medical offices and the BC Cancer Center - 9 - Mean 53.67 years - 8 females	- NA	- Lush green old growth forest	- 7–15 walks over 8 weeks, ≥150 min per week (moving time and distance was increased during the programme)	- In groups	Quantitative: - Generalized anxiety - State anxiety - Perceived stress - Sleep - Exercise self-efficacy - Self esteem - Well-being - Depression Qualitative: - Impact on psychosocial health (anxiety, well-being)
- [47] - D’souza - 2020 - India	- Quantitative (self-completion questionares), longitudinal	- Through trekking organizations - 58 - NA - NA	- Participants ranged from being beginners to expert trekkers	- In nature, in Bangalore, India	- From 2 to 6 days	- In groups	- Stress
- [21] - Mau - 2021 - Denmark	- Qualitative (semi-structured interviews), retrospective	- Through chain sampling - 9 - Mean 69 years - 7 females	- NA	- In nature	- From day walks to multi-week walks	- Individually and in groups	- Personal transformations

**Table A2:** Overview of the potential relation between long-distance walking and mental health.

Reference	What Type of Influence (If Any) Did Long-Distance Walking Have on Mental Health?	Which Aspects of the Walk Was Proposed as Associated with This Effect?	Which Theoretical Explanation Was Proposed Regarding the Relation between Long-Distance Walking and Mental Health?
[36]	Long walkers had significantly higher life satisfaction scores than the less active short walkers and inactives, and were significantly cognitivly younger than inactives, but not short walkers.	Physically active people may have a more positive outlook on life and therefore be better equipped mentally to deal with health problems. Likewise, maintaining health may be part of feeling younger.	
[58]	Participants experienced five types of fear while hiking alone: the fear of getting hurt by another individual, the fear of accidental injury/life-threatening emergency, the fear of getting lost, the fear of wild animals and dogs, and the fear of theft of belongings left in one’s vehicle.		Flow theory
[43]	All walkers remained above neutral regarding enjoyment levels and generally experienced the end of their walk as a climatic high; experienced walkers rated their level of enjoyment consistently higher with little fluctuation.		
[15]	The walk elicited positive emotions, reduced the effects of life-stress, and promoted an increased sense of well-being and personal growth.	Several aspects were mentioned, including the physical nature of the challenge and the sense of testing one’s own resolve, the uplifting scenic beauty and wildlife, having the time to reflect in a relaxing environment, and walking with other people and meeting people during the walk.	Broaden-and-build theory and others
[54]	Trekking was associated with positive life satisfaction. The effects of trekking were higher compared to mountaineering/rock climbing and cycling.	Participating in any outdoor activity gives positive life satisfaction as it is a measure of freedom; realizing this makes the person socialized.	
[46]	Hiking reduced hopelessness and depression significantly during the hiking phase of the study. There was no significant effect on suicide ideation, but suicide ideation was significantly decreased during the hiking phase.		
[55]	Hikers experienced flow.	The experience of flow can be predicted through three restorative qualities of being away, fascination and compatibility, as well as the hiker’s level of specialization.	Recreation specialization and restorative environments
[48]	Long-distance walking facilitated processes of relief and disengagement from common stresses and problems in life; helped people find ways to resolve their issues and fostered enduring positive self-directed change.	Aspects highlighted in interviews include opportunities for reflection and reappraisal.	A number of theories within positive psychology
[49]	Findings indicated a massive directive agency, and the impact of culture and nature on how participants think and relate to the world.	Ethical aspects seem to impact nurturing registers in nature-places and underpin therapeutic affect.	Spinoza and others
[40]	Moderate hiking did not increase cytokines, previously associated with deteriorated suicidality.		
[37]	Participants experienced considerable immediate improvements in their well-being due to their hike. Improvements of well-being increased with the level of activity, from walkers to hikers and runners.	The restorative effects of nature combined with physical activities may have a synergistic effect on well-being.	Restorative environments/biophilia, “green exercise”
[53]	Participants, both normal weight and overweight/obese persons, improved their mood status, feelings, and personal views on exercise.		
[51]	The effect of the trek on body awareness and identity was profound; participants experienced a sense of owning their body, allowing it to become a source of strength, joy, and meaningfulness. Participants described being less absorbed by their multiple sclerosis.	The relatively extreme, positive lived-body experiences during the expedition was necessary to anchor the lived body as normal; being watched through the eyes of the other participants.	Phenomenology
[41]	Salivary cortisol decreased in all conditions; however, this occurred more after mountain hiking and treadmill walking than the sedentary control situation.	Effects seemed to be caused by the physical activity and not by environmental effects, as there were no differences between mountain hiking and treadmill walking.	
[35]	After hiking, positive affective states (activation, elation, and calmness) were increased, while negative affective states (fatigue and anxiety) were decreased, compared to the sedentary control situation. No significant effects were observed regarding depression, anger, and excitement.	The results can be explained by the mere exposure to nature, by the effects of physical activity, and by the interaction between these variables.	Psychophysiological stress recovery theory and theory on affective benefits of “green exercise”
[38]	Participants who hiked regularly showed a moderately improved quality of life, which was an enhancement compared with patients who did not participate in the hiking intervention.		
[57]	The walk helped people to reconsider their quality of life and their active behavior. The walk also provided experiences of independence, thus giving a new meaning to their aging, and the walk gave them time to discover themselves.		Maslow’s hierarchy of needs
[44]	The long-distance collective walkers had more intense experiences in well-being compared to individual or small-group walking activities.	Walkers gained well-being from their social interaction experience, individual experience, and environmental experience.	Therapeutic mobilities theory and Construal level theory
[42]	One bout of mountain hiking is effective in influencing affective states positively and decreasing cortisol concentration. These changes were comparable when hiking in environments with less and more anthropogenic elements. Anthropogenic elements in the natural environment may play a minor role in affective states and stress-related physiological response.	Undetected stimuli of the natural environment (as opposed to a negative influence of anthropogenic elements) might explain the benefits of physical activity in a natural environment.	Attention Restoration Theory, Stress Reduction Theory, etc.
[45]	Hiking the trail did not affect participants’ well-being. However, the hike did increase well-being when the participant began with autonomous motivation or developed it over the course of the hike.		
[52]	The hike had a powerfull effect on the participants’ lived-body experience. They experienced being brought back to basics, a change in their bodily attunement, and a strengthening of their self-belief and social resilience.	Among other aspects, being close to nature and acquiring a more natural lifestyle, e.g., a daylight-based rhythm.	Body awareness theories
[50]	Most participants reported lasting effects of the walk, which were perceived to influence daily life, behavior, and future actions. These were mainly related to improved mental, physical, spiritual, and social health, greater personal health assets, and a more positive outlook on life.	The therapeutic mechanisms may lie in the physical effort, in a nature and social context.	Several theories within a salutogenetic perspective
[56]	Small, significant increases were found in the total score and subscale scores of a mindfulness questionnaire.	The increases may be explained by the apparent overlap of the contemplative and psychological processes operating during both the pilgrimage and mindfulness/meditation practices.	
[39]	Regarding quantitative findings, there were no significant effects in generalized anxiety, psychological well-being, depression, sleep, exercise self-efficacy, or self-esteem; however, a significant reduction in perceived stress and state anxiety were achieved. Regarding qualitative findings, psychological benefits were experienced, including anxiety alleviation and connection with nature.	The psychological benefits of physical activity and the restorative effects of exposure to nature may have a synergistic effect on mood, stress, anxiety, etc.	Restorative environments, “green exercise”
[47]	Participants with beginner and intermediate levels of trekking experience were less stressed immediately after the trek; experienced trekkers remained constant.		Theories related to the restorative potential of nature exposure and theories of stress
[21]	Participants experienced a sense of capability and that they had time to pursue emotionally difficult experiences. They also experienced how their reflections revolved around themselves and that they had an embracing approach to their thoughts.	The mental and physical strain, the simplicity in obligations, the solitariness and calmness of long-distance walking.	Liminality

## Appendix E. PRISMA Checklist

Preferred Reporting Items for Systematic reviews and Meta-Analyses extension for Scoping Reviews (PRISMA-ScR) Checklist.

**Table:**

**Section**	**Item**	**Prisma-ScR Checklist Item**	**Reported on Page Number**
**TITLE**			
Title	1	Identify the report as a scoping review.	1
**Abstract**			
Structured summary	2	Provide a structured summary that includes (as applicable): background, objectives, eligibility criteria, sources of evidence, charting methods, results, and conclusions that relate to the review questions and objectives.	1
**Introduction**			
Rationale	3	Describe the rationale for the review in the context of what is already known. Explain why the review questions/objectives lend themselves to a scoping review approach.	2–3
Objectives	4	Provide an explicit statement of the questions and objectives being addressed with reference to their key elements (e.g., population or participants, concepts, and context) or other relevant key elements used to conceptualize the review questions and/or objectives.	3
**Methods**			
Protocol and registration	5	Indicate whether a review protocol exists; state if and where it can be accessed (e.g., a web address); and if available, provide registration information, including the registration number.	3
Eligibility criteria	6	Specify characteristics of the sources of evidence used as eligibility criteria (e.g., years considered, language, and publication status), and provide a rationale.	3–4
Information sources *	7	Describe all information sources in the search (e.g., databases with dates of coverage and contact with authors to identify additional sources), as well as the date the most recent search was executed.	3–4
Search	8	Present the full electronic search strategy for at least 1 database, including any limits used, such that it could be repeated.	12
Selection of sources of evidence †	9	State the process for selecting sources of evidence (i.e., screening and eligibility) included in the scoping review.	3–4
Data charting process ‡	10	Describe the methods of charting data from the included sources of evidence (e.g., calibrated forms or forms that have been tested by the team before their use, and whether data charting was done independently or in duplicate) and any processes for obtaining and confirming data from investigators.	4
Data items	11	List and define all variables for which data were sought and any assumptions and simplifications made.	3–4
Critical appraisal of individual sources of evidence §	12	If done, provide a rationale for conducting a critical appraisal of included sources of evidence; describe the methods used and how this information was used in any data synthesis (if appropriate).	NA
Synthesis of results	13	Describe the methods of handling and summarizing the data that were charted.	4
**Results**			
Selection of sources of evidence	14	Give numbers of sources of evidence screened, assessed for eligibility, and included in the review, with reasons for exclusions at each stage, ideally using a flow diagram.	5
Characteristics of sources of evidence	15	For each source of evidence, present characteristics for which data were charted and provide the citations.	14–17
Critical appraisal within sources of evidence	16	If done, present data on critical appraisal of included sources of evidence (see item 12).	NA
Results of individual sources of evidence	17	For each included source of evidence, present the relevant data that were charted that relate to the review questions and objectives.	14–21
Synthesis of results	18	Summarize and/or present the charting results as they relate to the review questions and objectives.	5–8
**Discussion**			
Summary of evidence	19	Summarize the main results (including an overview of concepts, themes, and types of evidence available), link to the review questions and objectives, and consider the relevance to key groups.	8
Limitations	20	Discuss the limitations of the scoping review process.	9–10
Conclusions	21	Provide a general interpretation of the results with respect to the review questions and objectives, as well as potential implications and/or next steps.	10
**Funding**			
Funding	22	Describe sources of funding for the included sources of evidence, as well as sources of funding for the scoping review. Describe the role of the funders of the scoping review.	10

JBI = Joanna Briggs Institute; PRISMA-ScR = Preferred Reporting Items for Systematic reviews and Meta-Analyses extension for Scoping Reviews. * Where *sources of evidence* (see second footnote) are compiled from, such as bibliographic databases, social media platforms, and websites. † A more inclusive/heterogeneous term used to account for the different types of evidence or data sources (e.g., quantitative and/or qualitative research, expert opinion, and policy documents) that may be eligible in a scoping review, as opposed to only studies. This is not to be confused with *information sources* (see first footnote). ‡ The frameworks by Arksey and O’Malley (6) and Levac and colleagues (7) and the JBI guidance (4, 5) refer to the process of data extraction in a scoping review as data charting. § The process of systematically examining research evidence to assess its validity, results, and relevance before using it to inform a decision. This term is used for items 12 and 19 instead of “risk of bias” (which is more applicable to systematic reviews of interventions) to include and acknowledge the various sources of evidence that may be used in a scoping review (e.g., quantitative and/or qualitative research, expert opinion, and policy document). *From:* Tricco AC, Lillie E, Zarin W, O’Brien KK, Colquhoun H, Levac D, et al. PRISMA Extension for Scoping Reviews (PRISMA-ScR): Checklist and Explanation. *Ann Intern Med.* **2018**, *169*, 467–473, doi:10.7326/M18-0850.
